# Ultra-long carrier lifetime in neutral graphene-hBN van der Waals heterostructures under mid-infrared illumination

**DOI:** 10.1038/s41467-020-14714-1

**Published:** 2020-02-13

**Authors:** P. Huang, E. Riccardi, S. Messelot, H. Graef, F. Valmorra, J. Tignon, T. Taniguchi, K. Watanabe, S. Dhillon, B. Plaçais, R. Ferreira, J. Mangeney

**Affiliations:** 1 Laboratoire de Physique de l’Ecole Normale Supérieure, ENS, Université PSL, CNRS, Sorbonne, Université, Université de Paris, 75005 Paris, France; 2 State Key Laboratory of Precision Spectroscopy, East China Normal University, 200062 Shanghai, China; 3 Advanced Materials Laboratory, National Institute for Materials Science, Tsukuba, Ibaraki 305-0047 Japan

**Keywords:** Optical properties and devices, Electronics, photonics and device physics, Terahertz optics

## Abstract

Graphene/hBN heterostructures are promising active materials for devices in the THz domain, such as emitters and photodetectors based on interband transitions. Their performance requires long carrier lifetimes. However, carrier recombination processes in graphene possess sub-picosecond characteristic times for large non-equilibrium carrier densities at high energy. An additional channel has been recently demonstrated in graphene/hBN heterostructures by emission of hBN hyperbolic phonon polaritons (HPhP) with picosecond decay time. Here, we report on carrier lifetimes in graphene/hBN Zener-Klein transistors of ~30 ps for photoexcited carriers at low density and energy, using mid-infrared photoconductivity measurements. We further demonstrate the switching of carrier lifetime from ~30 ps (attributed to interband Auger) down to a few picoseconds upon ignition of HPhP relaxation at finite bias and/or with infrared excitation power. Our study opens interesting perspectives to exploit graphene/hBN heterostructures for THz lasing and highly sensitive THz photodetection as well as for phonon polariton optics.

## Abstract (web summary)

Long carrier lifetimes are beneficial for graphene-based optoelectronics, but carrier recombination processes in graphene possess sub-picosecond characteristic times. Here, the authors report carrier lifetimes ~30 ps at low energy in graphene/hBN Zener-Klein transistors, attributed to interband Auger processes.

## Introduction

Graphene/hBN heterostructures uniquely combine high-quality graphene owing to the use of encapsulating layers^1^ with the coupling of graphene carriers with the hyperbolic phonons in the hBN layers. Importantly, for applications they keep their valuable performance up to room temperature. Graphene/hBN heterostructures are very attractive for the development of interband semiconductor laser schemes at THz frequencies owing to the gap-less electronic band structure of graphene^2,3^ and the fine low-doping control. For the realization of a THz laser, carrier lifetimes of a few tens of picoseconds are needed to reach long-lived optical gain. In addition, sensitive THz photodetectors are achievable in graphene/hBN heterostructures owing to the high carrier mobility in encapsulated graphene and its broad absorption spectrum^4,5^. For a compromise between sensitivity and speed in such THz photoconductors, carrier lifetimes of a few tens of picoseconds are also required. More generally, the recombination dynamics of non-equilibrium carriers close to the Dirac point in the encapsulated graphene layer plays a crucial role in the performance of interband optoelectronic THz devices and recombination times of at least a few tens of picoseconds are often highly desirable^5–8^.

The recombination dynamics of non-equilibrium carriers in graphene has been widely investigated^9–12^. Mostly, femtosecond visible or near infrared pulses are used to generate hot non-equilibrium carriers of large density at high energies. After photoexcitation, the electron/hole populations are both redistributed (by energy-conserving intraband carrier–carrier scattering) and relaxed (by intraband electron–optical phonon scattering) within ∼50 fs. It results in two independent lower-energy broad electron and hole distributions, for which efficient interband Auger recombination processes leads to a single Fermi–Dirac distribution for conduction and valence populations within 100–200 fs. A subsequent cooling process occurs mediated by interband optical phonon emissions within 1–2 ps and by less efficient intraband acoustical phonon scattering^10,13^. Very recently, an additional recombination channel with picosecond decay time has been demonstrated in hBN encapsulated graphene relying on the coupling of hot carriers in graphene with hyperbolic phonon polaritons (HPhPs) in the hBN layers^14,15^. Few studies have used optical pump with energies lower than optical phonon energy and reported significant slowing of the carrier relaxation^16^. However, owing to the excitation of a large density of hot carriers, optical phonon emission remained the predominant relaxation channel^11,17^. In spite of intensive work, the investigation of recombination dynamic for carriers at low photon energy and fluence remains elusive in graphene, notably under dc bias control.

Here we use continuous mid-infrared laser excitation (ℏ*ω* = 117 meV) and set graphene at charge neutrality point (CNP) where transport is dominated by interband Zener tunneling^18,19^. This provides weak incident photon density and corresponds to a photon energy between the Fermi-level fluctuations (typically ~20 meV in graphene/hBN heterostructures) and the optical phonons of graphene and hBN (ℏΩ ≈ 170–200 meV). Focusing on this low-energy domain, we unveil on a strong photoconductive regime involving a remarkably long Auger recombination time (**~**30 ps) and a switching to a short HPhP emission time at a finite dc bias or mid-infrared optical power. It also allows to investigate the interplay between optical and electrical pumping.

## Results

### Graphene/hBN Zener–Klein phototransistor

**Fig. 1:**
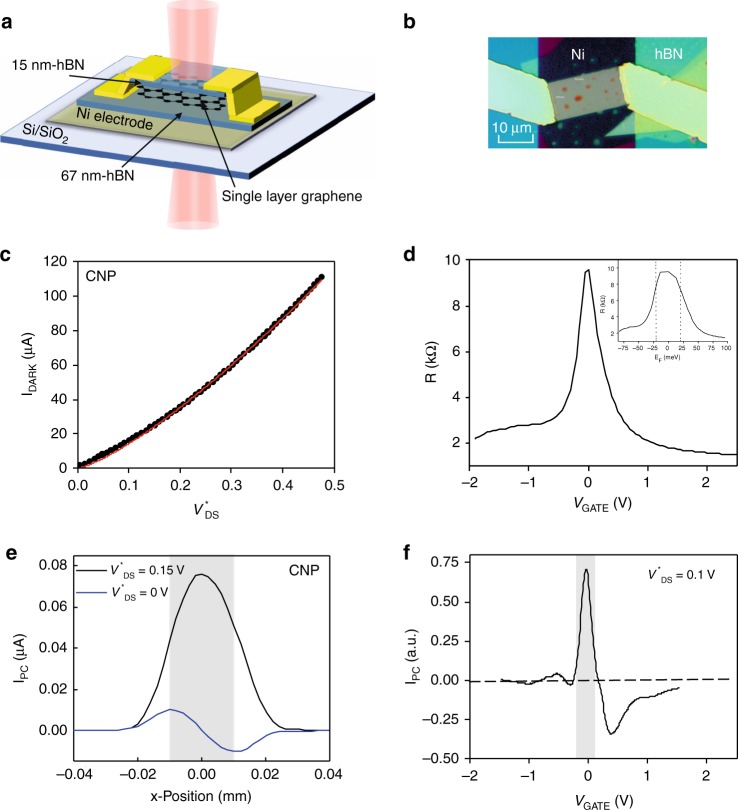
Graphene/hBN Zener–Klein phototransistor. Schematic drawing (**a**) and optical image (**b**) of the hBN-encapsulated graphene device with a semi-transparent Ni bottom gate electrode. **c** Dark-current bias characteristic (black square symbols) measured at 4 K and at the CNP (drain-doping effect is corrected by biasing the hBN/graphene heterostructure along constant density lines), with $V_{{\rm{DS}}}^ \ast = V_{{\rm{DS}}} - 2R_{\rm{c}}I_{{\rm{dark}}}$. The Zener–Klein tunneling current with *α* = 1.3 is shown as red dashed line. **d** Low-bias resistance measured at 4 K and *V*_DS_ = 10 mV; a carrier mobility of 3.2 m^2^/Vs, a residual carrier density of *n*_0_ = 4 × 10^10^ cm^−2^ ascribed to residual charge puddles, and a contact resistance *R*_c_ = 650 Ω are extracted. Insert: Low-bias resistance as a function of the Fermi-level energy using the relation $E_{\rm{F}} = \hbar v_{\rm{F}}\sqrt {\pi n}$ with $n = \sqrt {(C_{\rm{g}}\left| {V_{{\rm{GATE}}}} \right|/q)^2 + n_0^2}$. The gate capacitance, which mainly arises from the hBN dielectric capacitance with a negligible serial quantum capacitance, enables to access the Fermi energy range *E*_F_ ~ ±80 meV. The dashed lines represent the fluctuations of the Fermi-level energy induced by residual carrier density $E_{{\rm{F}}0} = \hbar v_{\rm{F}}\sqrt {\pi n_0}$ = 23.3 meV. At the carrier photoexcitation energy of 58 meV, the gate-induced net carrier density is significantly larger than the residual charge carrier density so that the estimation of the carrier mobility by the fit is valid. **e** Photocurrent line scan profiles of the graphene/hBN heterostructure along the graphene channel for $V_{{\rm{DS}}}^ \ast = 0\,{\rm{V}}$ (blue line) and $V_{{\rm{DS}}}^ \ast = 0.15\,{\rm{V}}$ (black line) under continuous light excitation at 10.6 µm wavelength measured at 4 K and at the CNP. The laser spot diameter is focused on the device with a waist of 21.2 µm quasi-matching sample length. The gray shadow area represents the graphene sample. **f** Photocurrent as a function of gate voltage for an illumination at the center of the channel and $V_{{\rm{DS}}}^ \ast = 0.1\,{\rm{V}}$. It shows a narrow peak with a half-width at half maximum of ~33 meV. The gray shadow area represents gate range of interest in this work.

We investigate a transistor (Fig. 1a) fabricated in an hBN-encapsulated exfoliated single-layer graphene (Fig. 1b). The hBN/graphene heterostructure, of dimensions *L* × *W* = 20 µm × 10.4 µm, is deposited on a Ni bottom gate electrode and equipped with Cr/Au edge contacts (see details in the ‘Methods’ section). The bottom hBN layer, of thickness 67 nm, limits electrostatic doping to the range ±0.15 × 10^12^ cm^−2^. The thickness of the Ni layer is set to 12 nm to be semi-transparent to mid-infrared light with a measured transmission of *T* = 10.9%. From low-bias resistance measurements at 4 K reported in Fig. 1d, we extract a carrier mobility of 3.2 m^2^/Vs, a residual carrier density of *n*_0_ = 4 × 10^10^ cm^−2^ ascribed to residual charge puddles due to disordered potential likely created by air gaps at the Ni–hBN interface, and a contact resistance *R*_c_ = 650 Ω. As discussed in Supplementary Note 1, the extraction of carrier mobility from gate-dependent resistance measurement is valid at energy close to the carrier photoexcitation energy of 58.5 meV since the fluctuations of the Fermi-level energy induced by residual carrier density are lower than $E_{{\rm{F}}0} = \hbar v_{\rm{F}}\sqrt {\pi n_0}$ = 23.3 meV^20^.

The dark-current bias characteristics were initially investigated. As discussed below, it is essential in this experiment to keep a low (constant) doping; to this end, we compensate for drain doping by a careful calibration of the gate voltage at the CNP as function of *V*_DS_ (which follows *V*_CNP_ = Const. + 0.56 V_DS_)^19^. The dark current, *I*_dark_, at the CNP and at 4 K follows a superlinear bias dependence (see Fig. 1c, black square symbols), as reported in earlier experiments^18^. The main contribution to the current in the graphene channel is ascribed to interband Zener–Klein tunneling. Indeed, Zener–Klein current, which is due to the tunneling of electron carriers from the valence to conduction bands^19,21^, follows a power law, $I \propto V^\alpha$ with 1 < *α* ≤ 1.5 that fits well our data for *α* = 1.3 (red dashed curve in Fig. 1c). A low bias and a slight deviation are observed that we attribute to finite charge puddle doping. Note that the Zener–Klein tunneling current is equivalent to an electrical pumping of non-equilibrium electron–hole pairs within the energy window *ε* < ±20 meV, as measured by noise thermometry^19^. Our optoelectronic experiment, which combines dc electric field and mid-infrared excitation, uniquely benchmarks the electrical and optical pumping of hBN/graphene heterostructures and provides unique insight of their interplay.

Using carrier transport measurements under mid-infrared continuous light excitation (CO_2_ laser of wavelength *λ* = 10.6 µm, i.e. photon energy of 117 meV), we investigated the photoresponse of the graphene/hBN transistor at energies below the in-plane optical phonon energy branch (170–200 meV) of both graphene and hBN. The laser spot diameter is focused on the device with a waist of 21.2 µm quasi-matching the sample length. Figure 1e shows the spatial distribution of the photocurrent along the channel length at the CNP for $V_{{\rm{DS}}}^ \ast = 0\,{\rm{V}}$ (blue curve) and $V_{{\rm{DS}}}^ \ast = 0.15\,{\rm{V}}$ (black curve) with $V_{{\rm{DS}}}^ \ast = V_{{\rm{DS}}} - 2R_{\rm{c}}I_{{\rm{dark}}}$. At zero source-drain voltage, the photocurrent is antisymmetric with respect to the sample length with extrema in the immediate vicinity of the leads and a node at the center. It can be assigned to photocarriers generated at the contact junctions that are accelerated by built-in electric fields at the contact as a result of the work function mismatch between graphene and the contact metal. Indeed, the regions close to the metallic leads (over typically 1 µm) become photoactive owing to band-bending. Both photovoltaic and thermoelectric effects likely play role in this regime^22^. By contrast, the photocurrent at finite source-drain bias is symmetric with a maximum at the sample center. It has the same polarity as the dark current and its maximum value is seven times larger than the maxima in the unbiased condition. The spatial profile of the photocurrent is well described by the convolution of a constant photoresponse along the channel with the Gaussian profile of the laser beam (see Supplementary Note 2).

Let us discuss the competing mechanisms responsible for this photocurrent at finite source-drain bias in graphene^5^. When a source-drain bias is applied, the uniform doping can be rendered asymmetric with a gradually effective doping along the graphene channel leading to the existence of thermoelectric effects in graphene. However, since only a weak gradient of temperature is induced by the large laser spot, the photocurrent contribution due to thermoelectric effect is expected to be very weak and opposite in sign to the dark current. Similarly, bolometric current, which results in the transport change produced by heating associated with the incident electromagnetic radiation, shows a vanishing contribution at low electrostatic doping and is also opposite in sign to the dark current due to decreasing mobility with increasing temperature (Fig. 1f at |*V*_GATE_| > 0.24 V). As a result, the photoconductive effect, where photoexcited electrons and holes are accelerated in opposite directions by an electric field, dominates over alternative photocurrent mechanisms at the CNP and at finite source-drain bias. As mentioned above, the photocurrent that relies on photoconductive effect is strongly sensitive to electrostatic doping. This is illustrated in Fig. 1f, which shows the gate voltage dependence of the photocurrent for an illumination at the channel center. We show a sharp peak at charge neutrality (half-width ~33 meV), narrower than the resistance peak itself (Fig. 1d). This dominates the photoresponse in the full doping range, in particular the negative photothermal response at large doping. It constitutes the signal of interest of this work. Although observed in previous photo-transport experiments^23–25^, these were in a regime of high-photon energy (>0.7 eV).

In the following, we investigate successively three relevant photoconductive regimes: (i) the linear regime where both bias and optical power are low, (ii) the non-linear regimes where either power or bias exceed the linear photoconductive limit, and finally (iii) the full photoconductive response including all regimes.

### Linear photoresponse

**Fig. 2:**
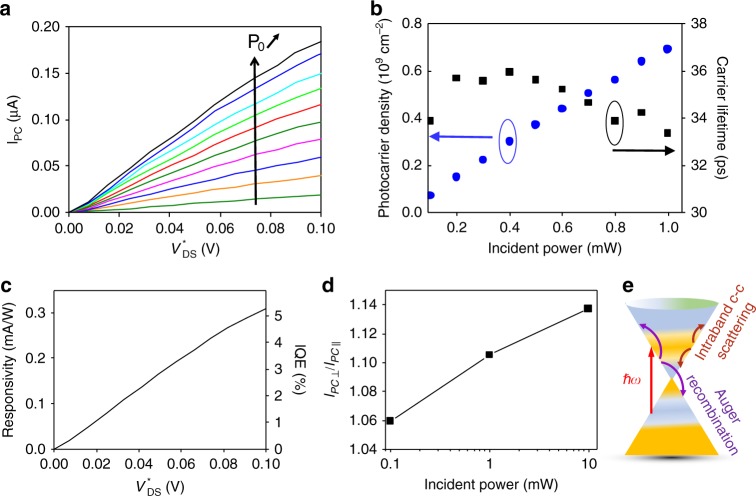
Graphene/hBN heterostructure phototransistor in the linear regime. **a** Linear photocurrent–bias characteristics measured at low bias for low incident power ranging from 0.1 to 1 mW. The measurements are performed at the CNP for a temperature of 4 K. The polarization of the incident light is linear, parallel to the dc electric field. **b** From photocurrent measurements and electrical characterization, we extract the photocarrier density and carrier lifetime as a function of the incident power in this low-bias regime, assuming negligible carrier multiplication effect (*M* = 1). Considering the layered geometry of our device and the size of the sample, the absorbed power *P*_abs_ = 6.8686 × 10^−4^ × *P*_inc_. **c** Responsivity and internal quantum efficiency (IQE) of the graphene/hBN heterostructure phototransistor as a function of $V_{{\rm{DS}}}^ \ast$. **d** Ratio between the photocurrent measured when light polarization is perpendicular to the dc electric field over the photocurrent when light polarization is parallel to the dc electric field as a function of *P*_inc_. **e** Sketch of the dominating relaxation and recombination processes: intraband electron–electron scattering and interband Auger recombination.

Figure 2a presents the photocurrent–bias characteristics at low-bias and low-incident mid-infrared power. The polarization of the incident light is linear, parallel to the graphene channel (and the dc electric field). It shows the linear dependence of the photocurrent with bias over a broad optical power range, as expected for a photoconduction process^23^. The photocurrent reaches the µA range. From these measurements and electrical characterization, we extract the photoexcited carrier density $n^{\ast}$ in this stationary regime at the CNP using *J*_PC_ = ${E_{DS}} q \mu n^{\ast}$ with *µ* = 3.2 m^2^/V/s and $E_{{\rm{DS}}} = \frac{{V_{{\rm{DS}}}^ \ast }}{L}$. The photoexcited carrier density reported in Fig. 2b as a function of the incident optical power evolves from <0.1 × 10^9^ to ~0.7 × 10^9^ cm^−2^. Note that the photoinduced electron and hole densities given by $n_{\rm{e}}^ \ast = n_{\rm{h}}^ \ast = n^ \ast /2$ are significantly higher than the thermal electron and hole densities (1.4 × 10^7^ cm^−2^) in ideal intrinsic graphene at *T* = 4 K. However, it remains relatively weak compared to the residual carrier density. To attain insight into the efficiency of the recombination processes, we can estimate the photoexcited carrier recombination time from the photocarrier densities and light excitation conditions^23^. The carrier diffusion is disregarded since the illumination and electric field are nearly uniform and the diffusion time is in the nanosecond range, i.e. much longer than recombination times. From a rate-equation approach, it follows that the total number of photoexcited carriers, with average density $n_{\rm{e}}^ \ast$, is given by $n_{\rm{e}}^ \ast = \alpha _0\frac{{P_{\rm{o}}}}{{S_{{\rm{laser}}}}}\frac{{M\tau }}{{\hbar \omega }}$, where *α*_0_ is the light absorption, *S*_laser_ the area of the laser spot, ℏ*ω* is the photon energy, *τ* is a phenomenological carrier lifetime, *M* is a carrier multiplication factor (resulting from impact ionization effect that can lead to the creation of additional carriers contributing to the photocurrent^26^) and *P*_o_ the laser power incident on the sample. *P*_o_ is lower than the incident optical power *P*_inc_ by a factor 0.63 due the larger size of the optical Gaussian beam with respect to the sample dimension. The light absorption *α*_0_ by the graphene layer is calculated using the transfer matrix method considering the layered geometry of our device (the hBN dielectric film of ~67 nm thickness and the Ni back gate of 12 nm thickness). We calculate the ratio of the electric fields at the graphene plane *E*_t_ and of the incident light *E*_0_ (see Supplementary Note 3). Then the graphene absorption is given by *α*_0_ = *A*(*E*_t_/*E*_0_)^2^ = 0.11% where *A* = 2.3% is the interband absorption in free space of monolayer graphene (in the absence of Pauli blocking at the CNP). We verify that the transmitted light through the device and the semi-transparent Ni electrode is proportional to the incident optical power, indicating no absorption saturation effect. In our experimental conditions, *M* is predicted to be close to unity. Indeed Jago et al. have calculated that *M* is typically <1.2 when photocarriers, with initial energy of 0.65 eV, evolve in a dc electric field^27^. Moreover, Tomadin et al. have predicted that *M* tends to unity when pump photon energy is lowered below 0.5 eV in undoped graphene sample^28^. Figure 2b shows the carrier recombination time (denoted carrier lifetime) extracted from this analysis, assuming negligible carrier multiplication effect (*M* = 1). We report on an unprecedented carrier lifetime of >30 ps (~35 ps in Fig. 2b), at low incident optical power and low bias, which is the main result of our work. This value is consistent with the slowing down of relaxation processes observed for low-energy carriers in graphene using pump–probe experiments, attributed to inefficient scattering via optical phonon emission^11,29^. In contrast to pump–probe experiments, which directly measure the dynamics of photoexcited carriers but with no direct distinction between relaxation and recombination processes, our experiment provides an estimation but singles out the recombination times of the photoexcited carriers. Indeed, only interband processes where electron and hole recombine are involved in the carrier lifetime *τ* since the intraband processes that relax the photocarriers within their respective band do not suppress the photocurrent.

Note that the carrier recombination times reported in Fig. 2b are underestimated if inhomogeneities of the Fermi level within the sample area due to charge puddles are considered since Pauli blocking reduces the effective absorbing area. Further, even if some carriers in the puddle region are taken into account in the calculation, the carrier lifetime still remains underestimated, as the mobility in the puddle region is lower than that in the linear region^30^.

This raises the question of the physical mechanism responsible for this very slow recombination of the photoexcited carriers. Recombination mechanisms of hot carriers in graphene usually involve the interplay between carrier–carrier and carrier–phonon scatterings. However, in this regime of low power, low bias and low temperature, scatterings via optical phonon in graphene and via coupling with hyperbolic phonon in hBN are strongly suppressed by Pauli blocking, as ℏ*ω* is well below their energy band 170–200 meV. With ℏ*ω* = 117 meV, one cannot rule out scattering by the lower 95–100 meV HPhP branch, a mechanism which is expected to be weak^31^. Acoustic phonon scattering cannot cause recombination because energy and momentum would not be conserved for interband transitions^32^. Indeed, the acoustic phonon mode, having an energy *ω*_*q*_ *=* *c*_*s*_*q* with *c*_*s*_ smaller than the graphene band velocity *v*_F_, does not provide a possible channel for interband transitions, which requires an energy greater than *v*_F_*q*. Emission of acoustic phonons is therefore only possible through intraband transitions^33^. The momentum mismatch issue can be circumvented by impurity-assisted phonon supercollisions^34,35^; however, this mechanism is suppressed in high-mobility graphene. Radiative emission is very slow in graphene, typically at nanosecond timescale^36^. Consequently, at low-bias and low-photoexcited carrier density, interband Coulomb scattering (Auger recombination) is expected to be the remaining scattering mechanism involved in the recombination of non-equilibrium carriers (i.e. photoinduced carriers in excess to the dark ones) in hBN/graphene/hBN transistors (see Fig. 2e). It is consistent with previous theoretical works predicting that interband Coulomb scattering times for carriers close to the Dirac point in graphene fall within the few to few tens of picoseconds range^36,37^. We attribute the independence of the carrier lifetime to the incident optical power observed in Fig. 2b to Auger recombination processes assisted by the large density of charges (Zener–Klein and residual carriers), which exceed the photoexcited carrier density at low incident power. As a result, the total charge density involved in Auger recombination process remains essentially constant from 0.1 to 1 mW incident optical power.

The responsivity and internal quantum efficiency of the graphene/hBN heterostructure transistor are shown in Fig. 2c. Despite the high carrier mobility and the long carrier lifetime in the encapsulated graphene layer, the responsivity and internal quantum efficiency (IQE) are relatively low compared to other reported mid-infrared graphene detectors^38^. The main reason is the weak photoconductive gain due to the long carrier transit time, in the nanosecond range, compared to the carrier lifetime, which results from the large drain-source distance of 20 µm. This length was set here to match the large diffraction limited spot size for mid-infrared light. However, advanced design such as antenna-assisted graphene detector, where optical antennas are used as both light-harvesting components and electrodes, can be implemented to greatly enhance simultaneously light-absorption and carrier-collection efficiency^39^.

For further insight into many-body Coulomb scattering, we take advantage of both the asymmetry of the photocurrent induced by the dc electric field and the asymmetry of the initial distribution of photoexcited carriers in *k*-space induced by the polarization of the incident light. Indeed, illuminating graphene with linearly polarized radiation provides an initial anisotropic distribution of photoexcited carriers in *k*-space with two lobes in the direction perpendicular to the light polarization. Figure 2d shows the ratio between the photocurrent measured when light polarization is perpendicular to the dc electric field over the photocurrent when light polarization is parallel to the dc electric field, indicating that the anisotropy of the photoexcited carrier distribution is not fully relaxed in this steady-state regime. This anisotropy is consistent with expectations even when considering that Coulomb scattering is predominantly collinear in graphene. As a matter of fact, non-collinear Coulomb scattering has been reported with a characteristic time of 2 ps^40^. Being much smaller than our recombination time, the polarization effect at the few percent scale agrees with our observation. The photocurrent and therefore the carrier lifetime are larger for perpendicular polarization, i.e. when the lobes of the photoexcited carriers are parallel to the shift induced by the dc electric field with respect to the Dirac point. In this configuration, Auger recombination processes are expected to be less efficient due to Pauli blocking by dark carriers, whose density is maximized in the direction of the dc electric field. In the following, we mainly use light polarization parallel to the dc electric field to minimize the interaction of photoexcited carriers with the out-of-equilibrium electron–hole pairs created by Zener–Klein tunneling.

### Non-linear photoresponse

**Fig. 3:**
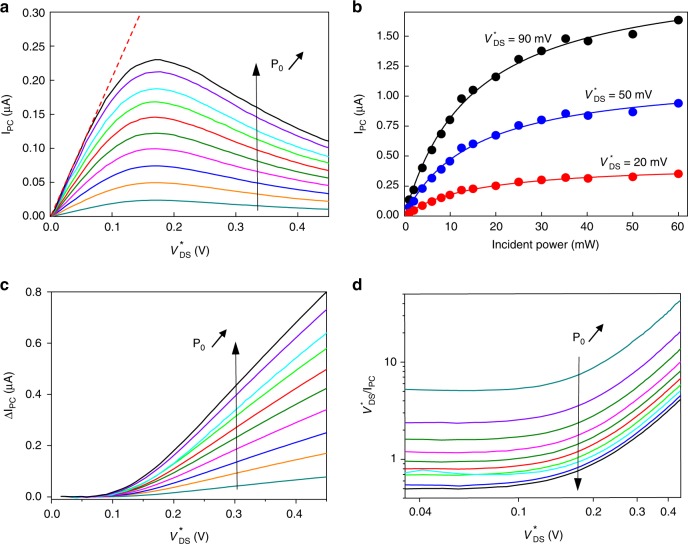
Graphene/hBN heterostructure phototransistor in the non-linear regime. **a**, **b** Photocurrent as a function of bias (**a**) and incident power (**b**) beyond the linear regime. The incident power remains low in **a**, ranging from 0.1 to 1 mW as well as the bias (20, 50, 90 mV) in **b**. The measurements are performed at CNP for a temperature of 4 K. The dashed line in **a** is the extrapolation of the linear regime $I_{{\rm{PC}}} = aP_{{\rm{inc}}}V_{{\rm{DS}}}^ \ast$. The plain lines in **b** represent standard saturation laws defined by $I_{{\rm{PC}}} = aP_{{\rm{inc}}}/(1 + P_{{\rm{inc}}}/P_{{\rm{sat}}})$, with a saturation power *P*_sat_ ~ 15 mW (*P*_abs_ ~ 10 µW) constant for all graphene bias. **c** Difference *ΔI*_PC_ between the photocurrent expected for photoconductive regime at large bias (dashed line in **a**) and the measured photocurrent as a function of $V_{{\rm{DS}}}^ \ast$ for *P*_inc_ ranging from 0.1 to 10 mW clearly showing a threshold behavior. **d** $V_{{\rm{DS}}}^ \ast /I_{{\rm{PC}}}$ as a function of bias in logarithmic scale for *P*_inc_ ranging from 0.1 to 10 mW; $V_{{\rm{DS}}}^ \ast /I_{{\rm{PC}}}$ is directly proportional to the total recombination rate *Γ*($V_{{\rm{DS}}}^ \ast$), itself the weighted sum of the Auger rate *Γ*_1_ and HPhP rate *Γ*_2_.

We now turn to non-linear regimes to further explore the hBN/graphene heterostructure-based devices. At low-incident optical power, we observe in Fig. 3a strong deviations from the $I_{{\rm{PC}}} \propto V_{{\rm{DS}}}^ \ast$ for $V_{{\rm{DS}}}^ \ast \ge 0.1\,{\rm{V}}$ leading even to a negative differential photoconductance for $V_{{\rm{DS}}}^ \ast \ge 0.17\,{\rm{V}}$, independently of the optical power in the range *P*_inc_ = 0.1–1 mW. The contrasted behavior between low bias and high bias cannot be described by the usual photoconductive effects^23^ and evidences the rise of an additional recombination channel for photoexcited carriers. The photocurrent drop, by more than a factor two, suggests that this additional recombination channel is highly efficient with a characteristic time significantly shorter than the low-bias recombination time of ~30 ps. To get a more accurate determination of the voltage threshold for the ignition of this additional channel, we plot in Fig. 3c the difference *ΔΙ*_PC_ between the extrapolated linear regime $I_{{\rm{PC}}} = aP_{{\rm{inc}}}V_{{\rm{DS}}}^ \ast$ (dashed red line in Fig. 3a) and the measured photocurrent. The obtained *ΔΙ*_PC_*(*$V_{{\rm{ds}}}^ \ast$) reveal a threshold at a voltage bias $V_{{\rm{ds}}}^ \ast = V_0 \le 0.1\,{\rm{V}}$, signaling the switching of the additional recombination channel. The value of *V*_0_, observed in two distinct samples, points to an activation energy close to energy of the upper HPhP band (ℏΩ_*II*_ = 170–200 meV) with *eV*_0_ ~ (ℏΩ_*II*_ – ℏ*ω*) ≈ 54–84 meV. We therefore attribute the additional recombination channel to the emission of HPhPs in hBN layer^41^, consistent with previous works on similar hBN-encapsulated graphene where picosecond HPhP cooling times were reported^14,42^. In particular, a similar threshold was observed in noise thermometric experiments using samples of similar mobility^19^. Indeed, h-BN supports large number of propagating HPhP modes (electromagnetic modes originating in the coupling of photons to optical phonons) that can be very efficiently coupled to the carriers in graphene via near-field coupling (i.e. super-Planckian coupling)^41^. These previous works have also demonstrated that recombination of electron–hole pairs in graphene through the direct coupling with intrinsic optical phonon in graphene (of comparable energy *ħΩ*_OP_ = 170–200 meV) plays a minor role in hBN/graphene heterostructures. This is because non-polar optical phonons are coupled to electrons via the deformation potential, giving rise to a smaller relaxation rate.

Figure 3d shows the bias dependence of $V_{{\rm{DS}}}^ \ast /I_{{\rm{PC}}}$, a quantity which is directly proportional to the total recombination rate *Γ*($V_{{\rm{DS}}}^ \ast$), itself the weighted sum of the Auger rate *Γ*_1_ and HPhP rate *Γ*_2_. *Γ*($V_{{\rm{DS}}}^ \ast$) increases strongly above the threshold *V*_0_ illustrating that *Γ*_2_ > *Γ*_1_. The gradual increase of *Γ*($V_{{\rm{DS}}}^ \ast$) for $V_{{\rm{DS}}}^ \ast \, > \, V_0$ indicates that electric field enables an increasing number of carriers within the relaxed carrier distribution to gain energy for HPhP emission. In this picture, *eV*_0_ is the onset energy for HPhP emission. The photocurrent maximum in Fig. 3a at $V_{{\rm{DS}}}^ \ast \sim 0.17\,{\rm{V}}$ is larger than *V*_0_ as a result of the combined effects of *Γ*_2_ > *Γ*_1_ and the broadening of energy/momentum carrier distribution. The relevance of a bias voltage criterion in governing the local carrier energy, *eV*_0_ ~ ℏΩ_*II*_ − ℏ*ω*, can be questioned in a diffusive regime. It is, however, supported by previous experiments using noise thermometry^19^. Our understanding relies on the carrier–carrier scattering rate (*τ* < 50 fs), dominating over impurity scattering rate (*τ* < 0.3 ps deduced from the mobility). In graphene, carrier–carrier scattering is prominently collinear (due to momentum/energy conservation), meaning that it preserves momentum direction with respect to electric field orientation. Considering that Fermi velocity is energy independent in single layer graphene, the effect of electric field in carrier acceleration is independent of energy relaxation and therefore reminiscent of a ballistic case. In this picture, the electric field (bias) dependence maps the photocarrier energy distribution with respect to the HPhP energy. Figure 3c, d shows that it is broadened while keeping track of the optical pumping energy in the onset threshold *eV*_0_ = ℏΩ − ℏ*ω* < 0.1 eV. The bias dependence thus provides an energy spectroscopy of the local carrier energy distribution.

As discussed in Supplementary Note 5, we can extract the photoexcited carrier density *Δn*_photo_ coupled to the HPhP, which falls in the range of 10^9^ cm^−2^ for *P*_opt_ = 1 mW, and the corresponding power drained away by HPhP emission given by *P*_HPhP_ = *Δn*_photo_ℏ*ω*_HPhP_/*τ*, which scales with the µW level.

Keeping low-bias conditions, the photocurrent shows non-linear behavior as a function of the optical power, above 1 mW, as depicted in Fig. 3b for $V_{{\rm{DS}}}^ \ast$=20, 50, 90 mV. We have verified independently that the absorption is still constant and does not saturate over this optical power range. The photocurrent obeys a standard saturation law $I_{{\rm{PC}}} = aP_{{\rm{inc}}}/\left( {1 + P_{{\rm{inc}}}/P_{{\rm{sat}}}} \right)$ (plain lines in Fig. 3b), with a *P*_sat_ ≈ 15 mW, independent of the graphene bias, corresponding to an absorbed optical power of 10 μW. To explain this non-linearity, let us consider that under large illumination, intraband carrier–carrier interactions (with ultrashort relaxation times of ∼20 fs) become more efficient, leading to a broadening of the hot carrier distribution within the electron and valence bands. Some fraction of hot carriers gain energy, potentially exceeding ℏΩ_*II*_, enabling some photoexcited electron–hole pairs to efficiently recombine in the hyperbolic optical phonon modes of the hBN layer. Consequently, we attribute the photocurrent saturation observed at high incident optical power to the enhancement of photoexcited carrier rates that efficiently recombine into HPhPs. As shown in Supplementary Note 4, this saturation effect is more pronounced for light polarization perpendicular to the dc electric field (i.e. for lobes of photoexcited carriers along the DC electric field). This is a result of out-of-equilibrium Zener–Klein carriers that provide both an increased number of available intraband Coulomb scattering partners to the photoexcited carriers as well as Pauli blocking at low energy. Thus the photoexcited carriers are redistributed towards higher energy states and their coupling to HPhP are enhanced. As discussed in the non-linear regime driven by large bias, we can also extract the photoexcited carrier density *Δn*_photo_ coupled to the HPhP (see Supplementary Note 5) and we find *Δn*_photo_ in the same range 10^9^ cm^−2^ with a similar threshold.

### Full photoconductive response including all regimes

**Fig. 4:**
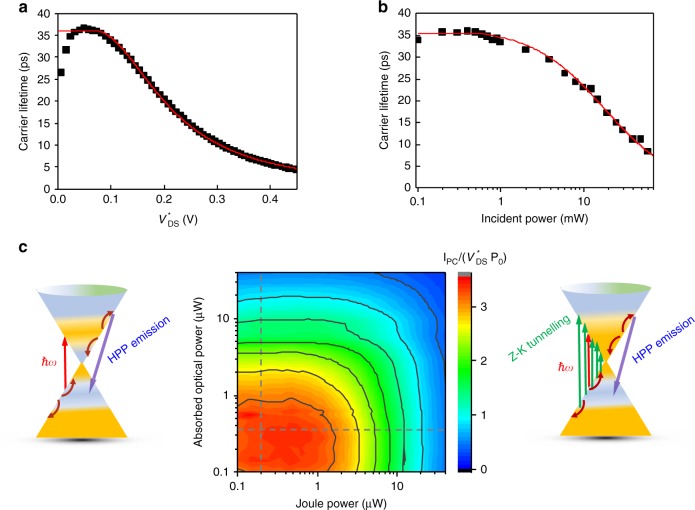
Electrical and optical pumping of graphene/hBN heterostructure transistor. Carrier lifetimes as a function of $V_{{\rm{DS}}}^ \ast$ (**a**) and *P*_inc_ (**b**) for *P*_inc_ = 0.5 mW and $V_{{\rm{DS}}}^ \ast = 0.1\,{\rm{V}}$, respectively, showing that both dc electric field and incident optical power act as distinct control knobs to switch on the non-linear response of the graphene/hBN heterostructure transistor resulting from the activation of the recombination channel via HPhP emission in the hBN layer. Red lines in **a**, **b** are theoretical fits of *τ* = *Γ*^−1^ with *Γ* = *Γ*_1_ + *Γ*_2_$\left( {V_{{\rm{DS}}}^ \ast - V_0} \right)^2$ and *Γ* = *Γ*_1_ + *Γ*_2_(*P*_inc_ − *P*_0_), respectively. A good agreement is found for *V*_0_ = 0.07 V, *P*_0_ = 0.5 mW, 1/*Γ*_1_ ≈ 35 ps, and a unique −1/*Γ*_2_ ≈ 0.7 ps for both fits. **c** Sketch of the dominating relaxation and recombination processes under high optical pumping at low bias (left) and under weak optical pump at large bias (left). The intraband carrier–carrier interactions become more efficient at high optical pumping leading to a broadening of the hot carrier distribution providing enough energies to some photoexcited electron–hole pairs to efficiently recombine through HPhP emission (right). Under large dc electric field and efficient carrier–carrier scattering owing to the presence of a large Zener–Klein carrier density, a fraction of carriers gains energies exceeding energy of 170 meV, enabling some photoexcited electron–hole pairs to efficiently recombine through HPhP emission (left). Center: Contour plot of the ratio of the photocurrent to the product of the bias times the incident optical power, $I_{{\rm{photo}}}/(V_{{\rm{DS}}}^ \ast P_{\rm{o}})$, as a function of the electrical Joule power $P_{{\rm{elec}}} = I_{{\rm{dark}}}V_{{\rm{DS}}}^ \ast$ and *P*_abs_ highlighting the interplay between optical and electrical pumping and the opto-electrical pumping of HPhPs in the hBN layer at high Joule power and high optical power. The two dashed lines represent the non-linear regimes of **a**, **b**.

Finally, we enlarge scope to the full photoconductive response including bi-non-linear effects. Figure 4a, b represent the carrier lifetimes as a function of $V_{{\rm{DS}}}^ \ast$ (at *P*_opt_ = 0.5 mW) and *P*_opt_ (at $V_{{\rm{DS}}}^ \ast$ = 0.1 V). They show that both $V_{{\rm{DS}}}^ \ast$ and *P*_opt_ act as distinct control knobs to switch on the pathway for hot carriers in graphene to recombine via HPhP emission in the hBN layer. Indeed, at low dc electric field and low optical power, the carrier lifetime is long, ~35 ps, and mainly governed by Auger recombination process, whereas we note that the ignition of the competing ultrafast HPhP recombination pathway is observed at large dc electric field and incident optical power. These results highlight the interplay between Auger recombination process and electron–hyperbolic phonon recombination channel. We deduce in Fig. 4a the characteristic time of the electron–hyperbolic phonon recombination process, corresponding to the carrier lifetime at very large bias, to picoseconds in full agreement with the picosecond hyperbolic cooling times measured in previous reports for hBN-encapsulated graphene^14,41^. This agreement confirms the validity of our method of carrier lifetime extraction. This two-recombination rate analysis is supported and quantified by theoretical fits of *τ* = *Γ*^−1^ in Fig. 4a, b (red lines) with *Γ* = *Γ*_1_ + *Γ*_2_$\left( {V_{{\rm{DS}}}^ \ast - V_0} \right)^2$ and *Γ* = *Γ*_1_ + *Γ*_2_(*P*_inc_ – *P*_0_), respectively. We deduce *V*_0_ = 0.07 V, *P*_0_ = 0.5 mW, 1/*Γ*_1_ ≈ 35 ps, and a unique −1/*Γ*_2_ ≈ 0.7 ps for both fits. Remarkably, the two different determinations of the threshold, from the onset in Fig. 3c on the one hand and by adjustment of large bias data in Fig. 4a on the other hand, give consistent values of the threshold voltage $V_0\sim \hbar ({\mathrm{\Omega }}_{II} - \omega )/e$.

Figure 4c summarizes the interplay between optical and electrical pumping. It shows a color plot of the full scope of these two methods of pumping the hBN/graphene heterostructures. The ratio $I_{{\rm{PC}}}/\left( {V_{{\rm{DS}}}^ \ast P_{\rm{o}}} \right) = A\mu \tau$ with $A = Wq\alpha _0/(L\hbar \omega S_{{\rm{laser}}})$ is represented as a function of the electrical Joule power $P_{{\rm{elec}}} = I_{{\rm{dark}}}V_{{\rm{DS}}}^ \ast$ and the absorbed optical power *P*_abs_. The linear regime appears as a red plateau; the non-linear regimes of Fig. 4a, b are represented by dashed lines. The symmetrical effects of electrical and optical powers are remarkably reflected in the color plot, including the first diagonal where both powers are comparably involved. This means that the photoresponse at charge neutrality merely measures the total number of excited carriers irrespective of their pumping pathway, presumably due to ultrafast intraband carrier–carrier scattering shuffling carrier energy. The non-linear regime at high Joule power and high absorbed optical power uniquely provides a true opto-electrical excitation of HPhPs. Combining mid-infrared illumination and large bias dc electric field in hBN/graphene heterostructure is very promising for developing source for HPhP optics. It promotes graphene/hBN heterostructures as a platform for studying the interplay between optical and electrical pumping of HPhP.

## Discussion

In conclusion, using mid-infrared photoconductivity measurements we have investigated recombination processes of carriers photoexcited at low density and energy in graphene/hBN Zener–Klein transistors. We have shown remarkable long carrier lifetime, ~30 ps, in quasi-intrinsic graphene, ultimately limited by interband Auger processes. Long carrier lifetime in graphene/hBN heterostructures could have important implications for THz lasing and highly sensitive THz photodetection. We have also unveiled the possibility to switch on at finite dc bias or mid-infrared optical power the very efficient electron–hyperbolic phonon recombination channel. This allows the carrier lifetime control, which falls below a few picoseconds upon ignition of HPhP relaxation. Investigating recombination processes for non-equilibrium carriers at low density and energy in alternative materials such as bilayer graphene and topological insulators could provide unique basic physic knowledge. Furthermore, we have investigated the interplay between optical and electrical pumping and demonstrated the opto-electrical pumping of HPhPs in the hBN layer at high Joule power and high optical power. These works could promote graphene/hBN heterostructures as a platform for phonon polariton optics and nanoscale thermal management.

## Methods

### Sample fabrication

The monolayer graphene and hexagonal BN flakes were produced by micro-mechanical exfoliation of bulk crystals on SiO_2_/Si substrates. We identified monolayer graphene sample by optical contrast and Raman spectroscopy, and clean hBN flakes were chosen after bright- and dark-field optical microscopy. Afterwards, we composed the hBN/graphene/hBN van der Waals heterostructures using a hot pick-up technique and we transferred the sandwich on the top of a 12-nm-thick Ni electrode. We localized the encapsulated graphene using Raman spectroscopy and we measured the hBN thickness and roughness using atomic force microscopy. After the sample characterization, we performed e-beam lithography on the heterostructure in order to define the design of the device (*W* × *L* = 10.4 µm × 20 µm) on the cleanest region of the sandwich and to obtain an HSQ resist mask. This mask was used to etch (Reactive Ion Etching) the top hBN layer in order to connect the graphene with one-dimensional edge contacts^43^. The last step of the device fabrication was the e-beam lithography to design the source, drain, and gate electrodes and metal evaporation (Cr-Au).

### Experiment

The experiment is based on a CO_2_ laser delivering 10.6 µm wavelength light modulated at 5 kHz. The laser light is focused on the sample using an aspheric Ge lens. The sample is placed within a liquid He cryostat with ZnSe optical window. The photocurrent signal is measured with a lock-in amplifier. A photovoltaic multiple junction detector is used to measure the mid-infrared light transmitted through the hBN/graphene heterostructure-based device.

## Supplementary information

Supplementary Information

## Data Availability

Statements of data availability and any associated references are available in Supplementary Information.
