# Supplementary material for: Ultra-long carrier lifetime in neutral graphene-hBN van der Waals heterostructures under mid-infrared illumination
Source: Nat Commun. 2020 Feb 13;11:863. doi: 10.1038/s41467-020-14714-1 (PMC7018796; doi:10.1038/s41467-020-14714-1)
Supplement: Supplementary file 1 — Supplementary Information [file 41467_2020_14714_MOESM1_ESM.pdf]

Ultra-long carrier lifetime in neutral graphene-hBN van der Waals heterostructures under mid-infrared illumination

P. Huang[1,2], E. Riccardi[1], S. Messelot[1], H. Graef[1], F. Valmorra[1], J. Tignon[1], T. Taniguchi[3], K. Watanabe[3], S. Dhillon[1], B. Plaçais[1], R. Ferreira[1], J. Mangeney[1]

[1] *Laboratoire de Physique de l'Ecole normale supérieure, ENS, Université PSL, CNRS, Sorbonne Université, Université de Paris, F-75005 Paris, France*
[2] *State Key Laboratory of Precision Spectroscopy, East China Normal University, Shanghai 200062, China*
[3] *Advanced Materials Laboratory, National Institute for Materials Science, Tsukuba, Ibaraki 305-0047, Japan*

Supplementary Note 1: Electrical Characterization

The gate-dependent resistance of the hBN/graphene based device is measured as shown on Supplementary Figure 1 (black curve). Considering the contribution of both electron and holes to the conductance of the graphene sheet, the total resistance of a device can be calculated as:

$$R = \frac{L}{W}\frac{1}{\sigma} + 2R_c = \frac{L}{W}\frac{1}{q(\mu_e n_e + \mu_h n_h)} + 2R_c \qquad (1)$$

with R_c the contact resistance of each electrodes and n, the local carrier density:

$$n = \sqrt{\left(\frac{\epsilon_0 \epsilon_{BN} |V_{GATE} - V_{DP}|}{e_{BN} q}\right)^2 + n_0^2} \qquad (2)$$

with ε_{BN}=3.2 and the bottom hBN thickness is e_{BN}=67 nm and n_0 is the residual charge density induced by puddles.

Using (1) to fit the measured gate-dependent resistance of the device (red curve in Supplementary Figure 1, we extract a contact resistance of R_c=650 Ω, a residual density n_0 of ~$4\text{x}10^{14}$ cm^{-2} and an electron and hole mobility of 3.2 m^2/V/s. The effect of residual density n_0 on transport behavior of graphene is impactive near the Dirac point whereas it is negligible far away from Dirac point. Indeed, extracting the carrier mobility from (1) is valid for gate induced net carrier density larger than the residual charge carrier density [S1]. This validates the estimation of carrier mobility from (1) close to the carrier photoexcitation energy of 58.5 meV since the fluctuation of Fermi level energy induced by residual carrier density are lower than $E_{F0} = \hbar v_F \sqrt{\pi n_0} = 23.3$ meV.

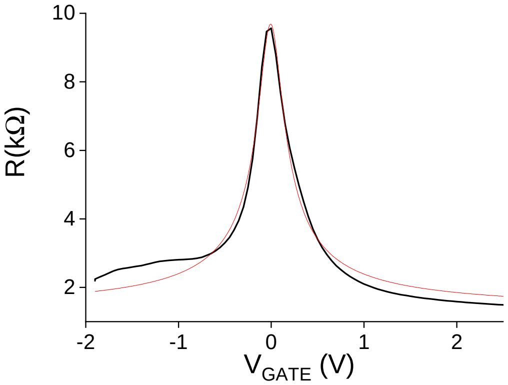

Supplementary Figure 1: Low-bias resistance measured at 4 K and V_{DS}=10 mV (black curve) and the fit using (1) (red curve).

Supplementary Note 2: Spatial profile of the photocurrent

From usual knife-edge measurements, we determine the beam waist of the optical gaussian beam at the sample position, w_0=10.6 µm. We compare the normalized spatial profile of the measured photocurrent (black curve in Supplementary Figure 2) with the convolution (red curve in Supplementary Figure 2) of a constant photoresponse along the graphene channel, given by a rectangular function $\prod(L)$, with the gaussian profile of the laser beam given by $P_{inc} = P_0 e^{-\frac{x^2+y^2}{2\sigma^2}}$ (with σ= 5.3 µm) and find a good agreement (without any adjustable

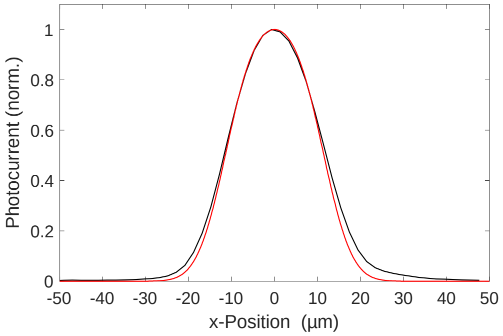

parameters).

Supplementary Figure 2: Normalized photocurrent line scan profile of the graphene/hBN heterostructure along the graphene channel for $V_{DS}^{*} = 0.15\,V$ (black line) under continuous light excitation at 10.6 µm wavelength measured at 4K and CNP. The red curve is the normalized convolution of a uniform photoresponse along the graphene channel given by a rectangular function with the gaussian profile of the laser beam P_{inc}.

Supplementary Note 3: Calculation of absorbed light power by graphene

The light absorption by the graphene layer is calculated considering the layered geometry of our device. We take into account the thickness and dielectric constants of each layer to find the electric field in the graphene plane. For this purpose, we use transfer-matrix method to calculate the electric field distribution within the layered structure. The light wavelength is 10.6 µm, the thicknesses of the top and bottom hBN dielectric films are ~67 nm and 15 nm respectively and the dielectric constant of hBN is 3.2. We have independently characterized the transmission of thin Ni films deposited on SiO_2/Si substrates to extract the dielectric constant of the 12 nm-thick Ni film and found ε_{NI}=9.06+37.87i, which is consistent with M. A. Ordal *et al.* [S2]. Supplementary Figure 3 shows the calculated spatial profile of the electric field E normalized by the incident electric field E_0 along the layered structure. The electric field at the graphene plane is $E_{Graphene} = 0.217E_0$. We deduce the graphene absorption given by $\alpha_0 = A(E_t/E_0)^2 = 0.11$ % where A=2.3 % is the interband absorption in free space of a monolayer graphene (since the Fermi level energy E_F is lower than $\hbar\omega/2$ at CNP).

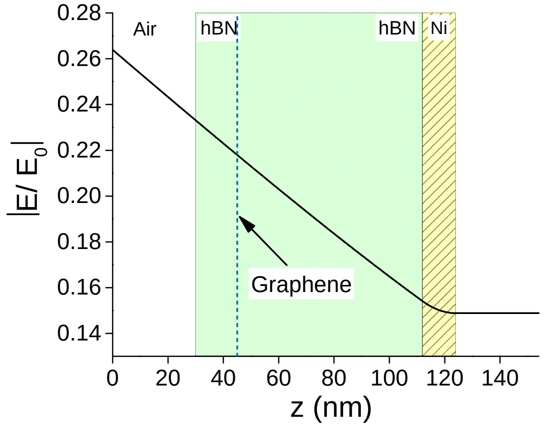

Supplementary Figure 3: Calculated electric field distribution along the stack of the hBN/graphene heterostructure using a transfer-matrix method. The dashed line represents the graphene layer.

Supplementary Note 4: Polarization dependence

We investigate how the nonlinear behavior of the photocurrent with the incident power depends on light polarization at low bias. The saturation effect is attributed to efficient intraband carrier-carrier scatterings under intense illumination that lead to a broadening of the hot carrier distribution within the bands. Thus, some photoexcited electron-hole pairs are shifted to energetically higher states where they efficiently recombine in the hyperbolic optical phonon modes of the hBN layer. As out-of-equilibrium Zener-Klein carriers provide both an increased number of available intraband Coulomb scattering partners to the photoexcited carriers and also Pauli blocking at low energy, intraband carrier-carrier scatterings and thus saturation effects are expected to be enhanced when the lobes of the photoexcited carriers are parallel to the dc electric field where the Zener-Klein carrier density is maximized, i.e. for light polarization perpendicular to the dc electric field. Our analysis is well supported by Supplementary Figure 4 that highlights a more pronounced photocurrent saturation effect with the incident power for light polarization perpendicular to the dc electric field.

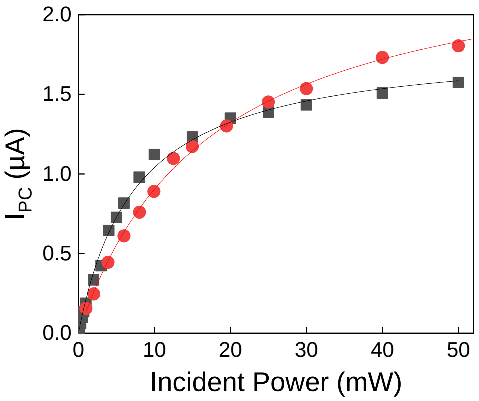

Supplementary Figure 4: Photocurrent measured for light polarization perpendicular (black square symbols) and light polarization parallel (red circle symbols) to the graphene channel and the dc electric field. Using the standard saturation law given by $I_{PC} = aP_{inc}/(1 + P_{inc}/P_{sat})$ (plain lines) we extract saturation powers of P_{sat}~15 mW and 7.5 mW for light polarization parallel and perpendicular to the graphene channel respectively.

Supplementary Note 5: HPhP emission in the two nonlinear regimes

We investigate the steady density of photoexcited electron–hole pairs supplied to the HPhP emission in the two nonlinear regimes. Applying (1) in the main manuscript, we extract in Supplementary Figure 5 (left) the photoexcited carrier density Δn_{photo} couple to the HPhP in the hBN layer at large V_{DS}^{*}, which falls in the range of 0.5 10^9cm^{-2} for P_{inc}=1 mW, and the corresponding power drained away by HPhP emission given by $P_{HPP} = \Delta n_{photo}\hbar\omega_{HPP}/\tau$ which scales with the μW level. At large P_{inc}, we also extract Δn_{photo} (see Supplementary Figure 5 (right)) from the difference between the photocarrier density expected for photoconductive regime at large incident power (dashed blue line) and the photocarrier density estimated from equation (1) in the main manuscript and measurements (blue circle symbols). The photoexcited carrier density Δn_{photo} that couple to HPhP in the hBN layer (square black symbols) is in the range of 10^9cm^{-2} and also follows a threshold behavior.

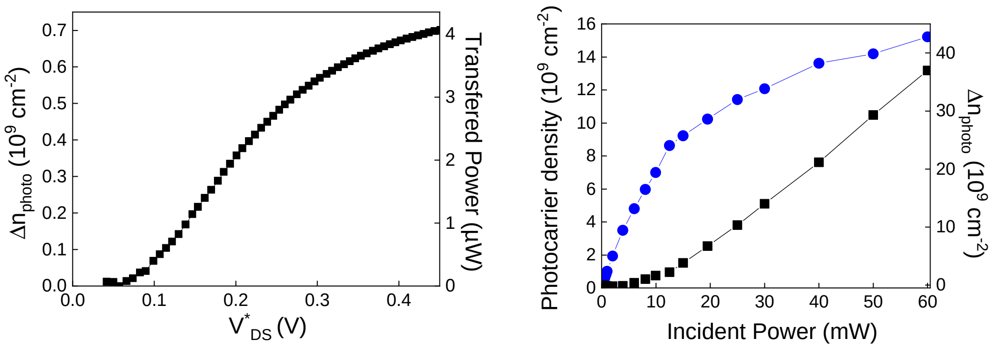

Supplementary Figure 5: Left: Photoexcited carrier density Δn_{photo} couple to the HPhP in the hBN layer and the power drained away by HPhP emission as a function of bias for P_{inc}=1 mW extracted from ΔI_{PC} using a rate-equations approach. Right: Photocarrier density, n_{photo}, as a function of the incident power extracted from electrical characterization (blue circles and left vertical axis) and Δn_{photo} (black squares and right vertical axis) the difference between the photocarrier density expected for photoconductive regime at large incident power (dashed blue line) and n_{photo} showing a threshold behavior with the incident power.

Supplementary references

[S1] Zhong, H., Zhang, Zhiyong, Xu, Haitao, Qiu, Chenguang & Peng, Lian-Mao, Comparison of mobility extraction methods based on field-effect measurements for graphene, AIP Adv. **5**, 057136 (2015)

[S2] Ordal M. A., et al. Optical properties of the metals Al, Co, Cu, Au, Fe, Pb, Ni, Pd, Pt, Ag, Ti, and W in the infrared and far infrared. Appl. Opt. **22**, 1099-1119 (1983)
